# Two Processes in Early Bimanual Motor Skill Learning

**DOI:** 10.3389/fnhum.2017.00618

**Published:** 2017-12-20

**Authors:** Maral Yeganeh Doost, Jean-Jacques Orban de Xivry, Benoît Bihin, Yves Vandermeeren

**Affiliations:** ^1^CHU UCL Namur, Stroke Unit/NeuroModulation Unit, Department of Neurology, Université catholique de Louvain, Yvoir, Belgium; ^2^NEUR Division, Institute of NeuroScience, Université catholique de Louvain, Brussels, Belgium; ^3^Louvain Bionics, Université catholique de Louvain, Louvain-la-Neuve, Belgium; ^4^Movement Control and Neuroplasticity Research Group, Department of Movement Sciences, KU Leuven, Leuven, Belgium; ^5^Scientific Support Unit, CHU UCL Namur, Université catholique de Louvain, Yvoir, Belgium

**Keywords:** bimanual coordination, motor skill learning, motor learning, inter-limb coordination, bimanual motor skill learning, motor coordination, robotics

## Abstract

Most daily activities are bimanual and their efficient performance requires learning and retention of bimanual coordination. Despite in-depth knowledge of the various stages of motor skill learning in general, how new bimanual coordination control policies are established is still unclear. We designed a new cooperative bimanual task in which subjects had to move a cursor across a complex path (a circuit) as fast and as accurately as possible through coordinated bimanual movements. By looking at the transfer of the skill between different circuits and by looking at training with varying circuits, we identified two processes in early bimanual motor learning. Loss of performance due to the switch in circuit after 15 min of training amounted to 20%, which suggests that a significant portion of improvements in bimanual performance is specific to the used circuit (circuit-specific skill). In contrast, the loss of performance due to the switch in circuit was 5% after 4 min of training. This suggests that learning the new bimanual coordination control policy dominates early in the training and is independent of the used circuit. Finally, switching between two circuits throughout training did not affect the early stage of learning (i.e., the first few minutes), but did affect the later stage. Together, these results suggest that early bimanual motor skill learning includes two different processes. Learning the new bimanual coordination control policy predominates in the first minutes whereas circuit-specific skill improvements unfold later in parallel with further improvements in the bimanual coordination control policy.

## Introduction

Most daily activities are bimanual, e.g., driving a car, typing on a computer keyboard, using a fork and a knife, and so on. These activities require a high level of coordination and motor planning as well as extensive practice and learning to be performed efficiently and smoothly with minimal effort. Insights regarding bimanual coordination cannot be fully gained by studying unimanual activities (Swinnen and Gooijers, 2015). Indeed, depending on the bimanual activity, various task requirements (sensori-motor, spatial, temporal or attentional) result in different levels of coordination complexity (Sleimen-Malkoun et al., 2011; Maes et al., 2017). For example, for rhythmic tasks, such as applauding, synchronized movements of the hands are recognized as “spontaneous” coordination patterns that rely on a preferential structural and/or functional organization of the central nervous system (CNS) (Kelso, 1984; Haken et al., 1985), i.e., a sort of “default-mode”. By contrast, other complex interlimb coordination patterns, such as typing with both hands, require learning and extensive training (Sailer et al., 2005; Sisti et al., 2011; Salimpour and Shadmehr, 2014).

Complex motor tasks are learned through repetition and training, which result in lasting improvements in the temporal and spatial accuracy of movements (Willingham, 1998; Shmuelof and Krakauer, 2011; Shmuelof et al., 2012). Overall, successful motor skill learning results in a shift in the speed/accuracy trade-off (SAT) and in a decrease in the inter-trial variability in performance (Dayan and Cohen, 2011; Shmuelof et al., 2012). Once acquired, skills can be generalized to other contexts (i.e., a new posture, a change in the scaling of movements, etc.). With sufficient training, consolidated skills can become “automatized,” i.e., be performed without requiring a substantial level of attentional load (Willingham, 1998), which allows individuals to allocate cognitive resources to other tasks.

According to Fitts and Posner (1967), motor skill learning consists of three stages: the first is a *cognitive stage* during which the subject tries to understand the task (i.e., its rules and fundamentals) and elaborate a strategy to accomplish the task; the second stage, *associative stage*, corresponds to the elaboration of the appropriate motor plan, which is refined over the course of trials; and the third and final stage is the so-called *autonomous stage* in which the learned behavior- the (sensori-)motor skill - is automatized, i.e., the skill is performed with a minimum of attentional resources. Another model of motor skill learning (Karni et al., 1998; Willingham, 1998; Doyon et al., 2003) suggests at least two phases. The first fast/early learning phase corresponds to sharp performance improvements that occur within the first session. This early phase is followed by a slow/late learning phase during which improvements occur over longer periods (days, weeks, etc.), are smaller in magnitude, and trend toward an asymptote. Whereas successive phases can be identified from a behavioral perspective (with somewhat arbitrary boundaries), recent theories suggests that several processes may operate in parallel to support motor skill learning. These processes may have different time courses or different “weights” over time (Clark and Ivry, 2010); that is, the different processes constituting a skill might be learned at different rates (Luft and Buitrago, 2005).

Behavioral studies have demonstrated that the fundamental basics of bimanual coordination are not the simple sum of those found for unimanual activities (Swinnen, 2002). Compared to unimanual motor skill learning, bimanual skill learning is more complex because an interlimb coordination control policy (i.e., a specific coordination pattern) needs to be acquired to synchronize the sequences of movements from both hands (Puttemans et al., 2005). When performing a task that requires a high level of interlimb coordination, like drumming, one first needs to establish the coordination control policy (Sailer et al., 2005). Thus, the need to develop a novel bimanual coordination control policy makes bimanual motor learning different from (and more demanding than) unimanual learning. As discussed by Swinnen (2002), the CNS seems to host some “default mode” for control policies for bimanual movements, such as in-phase (ø = 0°) or anti-phase (ø = 180°) movements in which the homologous muscles in both upper limbs are contracted either simultaneously (in-phase) or alternately (anti-phase). These “default mode” for coordination control policies play a structural role in learning the bimanual coordination control policies that require training to be performed efficiently (e.g., driving, playing the piano, etc.) (Maes et al., 2017).

Although the multiple neural processes that are interacting during the acquisition of a new unimanual skill have been studied extensively, these processes remain more elusive for bimanual learning. While learning a bimanual coordination control policy, most bimanual tasks impose various constraints, classified as neuromuscular, temporal or spatial (Swinnen and Gooijers, 2015). In healthy individuals who were learning a bimanual visuomotor task that consisted of applying forces with both hands to a manipulandum to hit targets, Sailer et al. (2005) identified three stages of bimanual motor skill learning: the first is the *exploratory phase* during which subjects build the primary mapping rules (i.e., understanding the structure of the skill and how to perform it in a given environment); the second is the *skill acquisition phase*, i.e., the time during which these rules are enforced; and the final phase is the *skill refinement phase* during which the movements become smoother.

To further explore the processes involved in the early (i.e., the first minutes) learning of a new bimanual coordination control policy, we designed a synergistic, physically coupled, non-rhythmic, bimanual task during which subjects drove a cursor along a complex path (called a circuit thereof) by coordinating their hands; this task is a bimanual version of the circuit game (Lefebvre et al., 2012a,b, 2015). Here, the left hand exclusively controlled the vertical motion of the cursor while the right hand exclusively controlled its horizontal motion. The shape of the complex circuit was designed so that coordinated bimanual movements were required to achieve the task. To separate the learning of the bimanual coordination control policy from that of the circuit-specific sequence (see below), we used a transfer test, i.e., we switched the circuit from one training block to the other at different stages of learning. We reasoned that improvements in the new bimanual coordination control policy would be insensitive to the circuit switch, while circuit-specific improvements would be more strongly influenced by the circuit used. Our hypothesis was that, during the first minutes, the new bimanual coordination control policy has to be established and learned, whereas task-specific (i.e., circuit-specific) aspects (i.e., the precise sequence of bimanual movements required to drive the cursor in a given circuit) would unfold later.

## Materials and Methods

### Participants

Fifty-one right-handed healthy individuals participated in the study after providing written informed consent. The study complied with the Declaration of Helsinki, and all procedures were approved by the Ethics Committee of the Université catholique de Louvain (UCL) (Approval number of ethical committee: 2011/15NOV/436). The inclusion criteria were being a right-handed healthy individual free of medication. The exclusion criteria were as follows: (1) a history of neurological problems, and (2) having less than 6 h of sleep the night before the experiment.

### Study Design

The bimanual coordination paradigm was implemented using an end-effector bimanual robot (Endpoint Kinarm, BKin Technologies, Kingston, ON, Canada). The subjects were seated comfortably and held the handles of the Kinarm in each hand. A complex circuit was displayed in the horizontal plane on the Kinarm’s monitor. The monitor provided visual feedback using a cursor but occluded direct vision of the hands/arms. The subjects learned the bimanual skill under different experimental conditions, all of which required the same bimanual coordination control policy. Both kinematics and dynamics were recorded at 1000 Hz and analyzed offline.

#### Bimanual Cooperation Paradigm

The learning paradigm was a bimanual version of the previously established unimanual circuit game (Lefebvre et al., 2012a,b, 2015). In the unimanual version of the circuit game, subjects were asked to navigate a cursor inside the circuit by means of a computer mouse controlled by their left hand (or paretic hand for stroke patients) as fast and accurately as possible. In the bimanual version, the cursor was navigated through the coordinated movements of both hands: the right hand controlled the horizontal displacement of the cursor, and the left hand controlled the vertical displacement. The movements of the right (left) hand were constrained to the horizontal (vertical) axis by virtual walls, i.e., forces exerted by the Kinarm on the handles of the robot that were defined in all non-desired directions for that handle. For example, for the right handle, these were forces in all directions except for horizontal movements.

**FIGURE 1:**
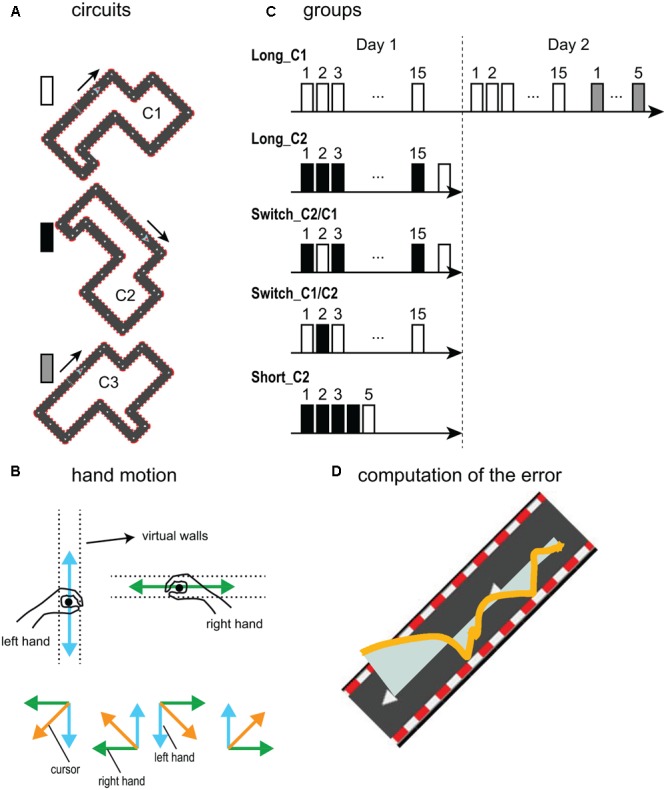
Experimental setup. **(A)** The three forms of the circuit experienced by the subjects: circuit 2 (C2) was the rotated form of circuit 1 (C1); circuit 3 (C3) had the same segments with a different order. **(B)** Order of presentation of the circuits for the five groups. The color of the bars indicates which circuit is used. **(C)** In this bimanual task, the right hand was restricted to move in horizontal axis and the left hand to vertical axis in the XY plane. Based on the orientation and angle (±45°) of each segment of the circuits, there are four possibilities for achieving successful coordinated bimanual movements: For the segments with 45° from the *X*-axis, (1) to ascend, subjects need to go right with the right hand and up with the left hand; (2) to descend, they need to go left with the right hand and down with the left hand, and for the segments with –45° from the *X*-axis, (3) to ascend, they need to go left with the right hand and up with the left hand; (4) to descend, they need to go right with the right hand and go down with the left hand. **(D)** The error was computed as the surface between the cursor trajectory and the ideal trajectory (light blue area), defined as the midline of the track. The orange line represents the real trajectory of the cursor.

All of the segments of the circuits were tilted 45° from the horizontal axis (**Figure 1A**); thus, coordinated bimanual movements were required to efficiently navigate the cursor. Because all of the circuit’s segments had an angle of ±45° with respect to the horizontal direction, equal contributions of both hands (each one exclusively controlling the vertical/horizontal displacement and thus equally angled at 45° with respects to the circuit’s segments) resulted in the ideal cursor motion. That is, staying on the midline of the circuit required the two hands to move at the same speed but along different axes (i.e., vertical or horizontal). Based on the forms of different segments in a circuit, there were four possibilities of such bimanual coordinated movements (**Figure 1B**) that the subjects needed to learn to correctly perform the task. Here, a bimanual coordination factor (Bi-Co, see below) was defined to quantify the bimanual coordination control policy in this cooperative, non-rhythmic and non-cyclic task. Many studies have used the “relative phase” to investigate bimanual coordination (Kelso, 1984; Rosenblum and Kurths, 1998; Sallagoïty et al., 2004; Lafe et al., 2016), i.e., the phase difference between two hands’ movements, which is an accepted optimum measurement for studying bimanual coordination in cyclic movements. However, our bimanual cooperative task required non-rhythmic and non-cyclic hand movements in different directions; thus, a measurement other than the relative phase was necessary to accordingly quantify the bimanual coordination. Therefore, the Bi-Co was computed as the normalized measure of the smallest value between the absolute values of the velocities of the two hands, thus, Bi-Co quantified how well the hands’ movements were coordinated. Because of the design of the bimanual circuit game, the ideal Bi-Co corresponded to the equal speed of both hands in different directions (see the Data Analysis).

The aim of the task was explained as follows: to move the cursor as fast as possible (i.e., to make as many turns as possible during each 1-min block) while staying inside of the circuit and ideally on the midline of the circuit. The ideal “accuracy” was defined as keeping the cursor in the midline of the circuit, and the error was calculated as the surface that is made between the trajectory of the cursor and the midline of the circuit (**Figure 1D**). Thus, this task involved a (bimanual) speed-accuracy trade-off (Bi-SAT). At the end of each block (1 min), an online score was displayed to provide global feedback about the subject’s performance.

Feedback score = time in the circuit during the lap/(time for a lap)^1.4^

This score was not used as an outcome measure and was only used to motivate the subjects.

#### Reaching Task

During the first phase of the study (only in *Long_C1* group), a reaching task was used. In this task, there was a home point with 5 different targets (5 circular targets, radius: 1 cm, positioned at 45°, 67.5°, 90°, 112.5°, and 135° with respect to the horizontal line and 10 cm distant from the starting position) that were displayed one-by-one in a randomized order. The subjects quickly reached from the home point to the target using the coordinated bimanual movements. The subjects were instructed to move the right hand along the vertical axis and the left hand along the horizontal axis. Importantly, in this task, there were no virtual walls so the hands were free to move in all directions. The goal was to investigate subjects’ performances in a task with the same rules (i.e., the right (left) hand controlled the horizontal (vertical) movements) but (i) without physical constraints (no virtual walls) and (ii) with the necessity to adjust the learned bimanual control policy based on each target position. That is, the 45° and 135° targets required the same bimanual control policy as did the circuit (ideally: equal speed for each hand), but the three others targets required different bimanual control policies, i.e., proportionally different speeds for each hand. Furthermore, some of the blocks were performed under and some without visual feedback. The bimanual reaching task was performed at the start and end of training with the C1 circuit on both days (group *Long_C1* only). Despite training with the virtual walls during the circuit game in reaching task, the subjects very quickly reverted to symmetrical reaching movements with both hands, a more “natural” or spontaneous bimanual control policy. Therefore, the data of reaching task were not considered for further analysis.

#### Circuits

The circuits C1 and C2 were identical in terms of the lengths and order of the different segments but C2 was rotated 90° clockwise compared to C1 (**Figure 1A**) and thus required a different *sequence* of combined bimanual movements. The circuits C1 and C3 were also made of identical segments, but with the four segments arranged in different orders while maintaining the same overall length (**Figure 1A**).

#### Experimental Protocol

The subjects (*n* = 51) were separated into five groups with slightly different designs (**Figure 1C**). Only the subjects in the *Long_C1* group trained over two days, the other groups trained over one day. All subjects began with 30 s of familiarization during which they became acquainted with the task environment using a 45°-rotated square to explore the requirements in four possibilities of bimanual coordination control policy.

##### Long_C1

In group *Long_C1*, the subjects (*n* = 11) were trained over two consecutive days during 15-min sessions. On Day 1, after familiarization, the subjects performed two baseline runs of the center-out reaching task, i.e., one run with and another run without visual feedback (40 targets /run). These subjects then learned the bimanual circuit game during 15 consecutive runs of 1 min each interleaved with 30 s of rest. Finally, they performed a post-learning run of reaching without visual feedback (40 targets). On Day 2, these subjects began with one run of reaching without visual feedback. Then, they were trained again on the bimanual task with the same protocol as on Day 1. Afterward, they performed two runs of reaching, one without and then one with visual feedback. Finally, they performed 5 blocks of 1 min on another circuit (C3) of the same length and difficulty but with a different shape so that we could evaluate transfer.

##### Long_C2

For the *Long_C2* subjects (*n* = 11), after familiarization, the training consisted of 15 blocks of C2 followed by one block of C1.

##### Switch_C1/C2 and Switch_C2/C1

After familiarization, the subjects in both groups trained with the two circuits (C1 and C2 switching). The *Switch_C1/C2* subjects (*n* = 10) began the first block with C1, trained on C2 during the second block, then C1 and C2 were switched until the 15th block (C1). The *Switch_C2/C1* subjects (*n* = 8) followed the same protocol except that they started with C2 and finished with an additional 16th block of C1.

##### Short_C2

The protocol for the *Short_C2* subjects (*n* = 11) consisted of 30 s of familiarization and 4 blocks of training with C2, followed by a final block of C1.

### Data Analysis

The data were analyzed off-line with custom-made MATLAB routines (MATLAB and Statistics Toolbox Release 2012b, The MathWorks, Inc., Natick, MA, United States). To quantify different aspects of cooperative bimanual motor skill learning, we defined 3 outcome measures that were quantified for each time point and then averaged for each 1-min block separately:

1- Bi-SAT, which was calculated based on the following formula:

$$\begin{array}{c} \mathrm{SAT}\;=\;C(Speed/Error)\;\\ (C = 1 cm.s) \end{array}$$

In which the constant C was defined to make the SAT measure dimensionless. The speed measure reflects the vectorial velocity of the cursor (cm/s) averaged over each 1-min block. The error was the surface (cm^2^) that was made between the cursor position and the mid-line of the track averaged over each 1-min block. Thus, the error was quantified as the surface resulting from the deviation from the ideal trajectory (i.e., the midline of the circuit). Note that the position and speed of the cursor result from the combination of the two hands’ movements, each along a different axis. Higher SAT values are associated with more accurate movements and/or higher speed.

2- The bimanual coordination factor (Bi-Co) is the normalized measure of the smallest value between the velocities of the two hands and was defined as follows:

$$\begin{array}{c} Bi-Co\;=\;\min(|V_{x}|,|V_{y}|)/||V|| \end{array}$$

Where:

min(|V_x_| ,|V_y_|) is the minimum value between |V_x_| (absolute value of the horizontal hand velocity) and |V_y_| (absolute value of the vertical hand velocity. This value was normalized by the hands’ vectorial velocity $\left(||V||=\sqrt{V_{x}^{2}{+V}_{y}^{2}}\right)$. There is a penalty for this measure when one hand is moving while the other is not. The Bi-Co ranged from zero (when one hand did not move) to 0.7 (when the two hands had exactly the same speed).

3- The forces exerted by the subjects in non-desired directions against the virtual walls were stored for analysis. Irrespective of how much the subjects pushed in non-desired directions, the virtual walls constrained each hand to slide along its imposed axis. These forces were expected to decrease as learning progressed. Bi-F was computed as follows:

$$\begin{array}{c} ||F||=\sqrt{F_{x,\mathrm{left}\;\mathrm{hand}}^{2}{+F}_{y,\mathrm{right}\;\mathrm{hand}}^{2}} \end{array}$$

Where:

||F|| is the magnitude of the force in non-desired directions. E.g., F_x,left hand_ is the component of the force exerted in the horizontal directions for the left hand (responsible for vertical cursor displacement), i.e., how much the left hand pushed against the horizontal virtual walls.

The data of the 3 outcome measures were computed at 1 kHz and then averaged for each block of training (1 min) per subject. The outcomes were then normalized with respect to the first minute of training (block 1,*Outcome*_1_). Therefore, the percent of change of an outcome for the block n (*P_n_*) was computed as follows:

$$\begin{array}{c} P_{n}=\frac{{\mathrm{Outcome}}_{n}-\;{\mathrm{Outcome}}_{1}}{{\mathrm{Outcome}}_{1}}\times 100\;\;\;\;\;(Equation1) \end{array}$$

where Outcome_n_ represents the value of one of the three outcomes for the block n.

To investigate the effect of training, improvements were quantified through their percent of change in the 15th block (for Day 1) and the 30th block (for Day 2) for *Long_C1* and in the 15th block for *Long_C2* (15 blocks of training). To test the overnight loss in *Long_C1* from Day 1 to Day 2, the loss in the first block of Day 2 compared to the last block of Day 1 was calculated. To quantify the loss in performance after switching the circuit at two different time points during the training (i.e., after 15 or 30 min) and to assess transfer between the circuit used for training (C1 or C2) and the new circuit (C3 or C1, respectively), we computed the changes between the last block of training [block EoL (End-of-Learning): block 30 for *Long_C1*, block 15 for *Long_C2*, block 4 for *Short_C2*] and the first block of transfer (block T1: block 31 for *Long_C1*, block 16 for *Long_C2*, block 5 for *Short_C2*). The decrement in performance due to the circuit change was computed as follows:

$$\begin{array}{c} P_{\mathrm{EoL}}=\frac{{\mathrm{Outcome}}_{\mathrm{EoL}}-{\mathrm{Outcome}}_{T1}}{{\mathrm{Outcome}}_{T1}}\times 100\;\;\;\;\;\;(Equation2) \end{array}$$

### Statistical Tests

#### Analysis 1

The goal of this analysis was to compare the drop in the outcomes when the circuit was suddenly changed to another circuit after learning during 15 (*Long_C2*) or 30 min (*Long_C1*) (*Equation 2*). A two-tailed t-test was used to compare these values against 0. This analysis explored the contribution of circuit-specific process after 15 and 30 min of training.

#### Analysis 2

The goal of this analysis was to test whether training with a single circuit was different from training with two switching circuits. We compared the performance of the four following groups on block 15: *Long_C1* (block 15: circuit C1), *Long_C2* (block 15: circuit C2), *Switch_C1/C2* (block 15: circuit C1), and *Switch_C2/C1* (block 15: circuit C2). There were two between-subject factors used for this ANOVA: the factor schedule (*Long vs. Switch*), and the factor starting circuit (*circuit C1 or C2*). Note that the starting circuit also determines which circuit was experienced on block 15. The 3 outcomes were submitted to this 2 × 2 ANOVA. This analysis explored whether the level of performance was affected by the schedule (*Long vs. Switch)* or the starting circuit (*circuit C1 or C2*).

#### Analysis 3

The goal of this analysis was to look for differences in performance between the two circuits that may have arisen later in learning. We used a repeated-measure ANOVA with circuit (*C1 or C2*) as the within-subject factor and group as the between-subject factor (*Switch_C1/C2 vs. Switch_C2/C1*). The outcomes were averaged for each circuit separately (blocks 9–11–13–15 vs. 8–10–12–14 blocks, depending on the group and circuit). This analysis explored whether the performance in both circuits reached the same level in both *Switch* groups.

#### Analysis 4

The goal of this analysis was to look for differences in loss of performance due to the transfer between the short (*Short_C2*) and long durations of training (*Long_C2*). Subjects from both groups were trained on C2, and transfer was tested on C1. For both groups, the decrement of performance due to transfer was computed as in *Equation 2* for the outcomes. The values for the two groups were compared with two-tailed *t*-tests. This analysis explored the difference between the loss due to the circuit switch after 4 and 15 min of training.

#### Analysis 5

To estimate the relationship between the outcomes, we looked for correlation coefficients. We grouped the data from the 40 subjects who experienced 15 min of training (i.e., *Long_C1, Long_C2, Switch_C1/C2* and *Switch_C2/C1*). For all these subjects, the normalized outcomes (*Equation 1*) were measured at block 15. This analysis explored whether the three outcomes followed a similar evolution (suggesting they captured a common process) or not (suggesting they reflected different processes).

All statistical tests were conducted using MATLAB R2015.

## Results

We asked our subjects to control a bimanual robotic manipulandum to guide a cursor across a complex circuit as fast and accurately as possible. The critical manipulation was that the two hands were controlling different dimensions of the cursor motion. The movement of the left (resp. right) was mechanically constrained in the vertical (resp. horizontal) dimension and controlled the vertical (resp. horizontal) motion of the cursor. That is, each hand made a specific contribution to the cursor motion, and because all the segments of the circuits were tilted, both hands had to move together to displace the cursor efficiently within the track.

**FIGURE 2:**
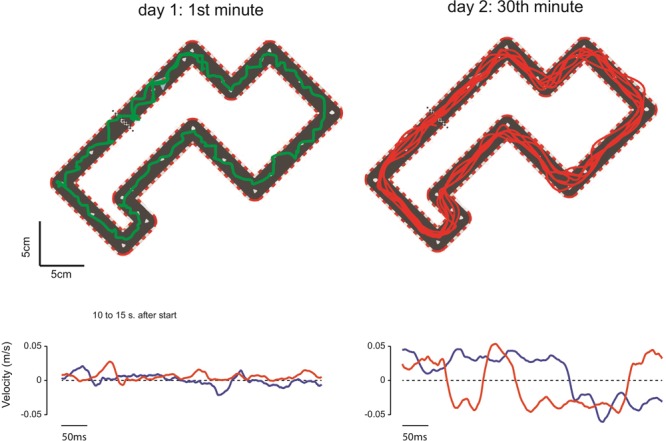
Behavior during the first and 30th minute of training. **(Top)** Cursor trajectory during the 1st minute of the training (left, green trace) and during the 30th minute of training (right, red trace). **(Bottom)** Velocities of the left (red) and right (blue) hands (sample of 5 s between 10 and 15 s after the go signal), bottom left: on the 1st minute of training; bottom right: on the 30th minute of training.

In the first minute of exposure (**Figure 2**, top left panel), the subjects typically moved very slowly and the trajectory of the cursor was irregular, highlighting the lack of bimanual coordination control policy. During this first block, the hands never moved concomitantly at a fast speed (lower left panel). In most cases, a peak velocity for one hand was accompanied by a minimum velocity (in absolute value) for the other hand. That is, the subject could not move both hands simultaneously at high speed. By contrast, the pattern of cursor motion was completely different after 30 min of training (top right panel). By the last block of training, the subjects were able to perform several laps of the circuit. The motion of the cursor was smoother because the two hands were well coordinated (lower right panel). After training, hand velocities approached the ideal coordination control policy, i.e., the two hands had the same speeds (see Materials and Methods).

### Training with a Single Circuit (Day 1)

**FIGURE 3:**
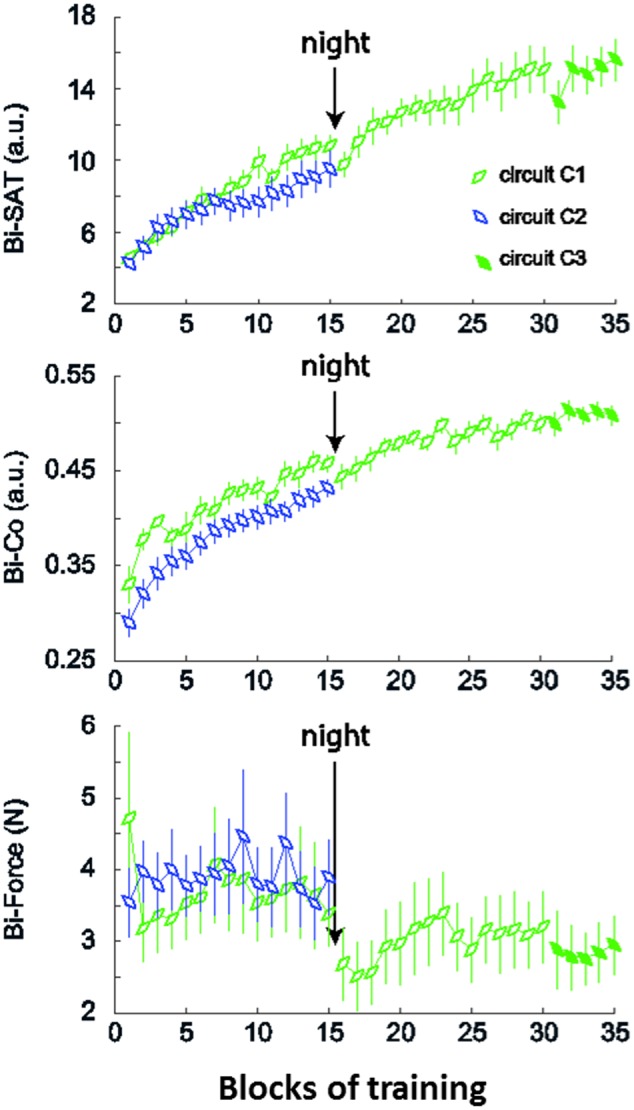
Evolution of the outcome measures for *Long_C1* and *Long_C2*. The plots demonstrate the evolution of the mean of the 3 outcome measures for *Long_C1* (in green) during the 2 days of training and *Long_C2* (in blue) during 1 day of training. The top plot shows the mean values of Bi-SAT; Bi-Co is in the middle plot and the lowest plot indicates the Bi-F. Error bars represent standard error of the mean (SEM).

We quantified the amount of improvement from the *Long_C1* and *Long_C2* groups on Day 1 (**Figure 3**) using three outcomes (*Equation 1)*. Subjects from both groups improved in terms of the speed/accuracy trade-off (mean (±SD, [95% CI]) for the Bi-SAT: *Long_C1* (Day 1): 148% (± 73%, [105% to 191%]); *Long_C2*: 126% (±45%, [99% to 153%])) from block 1 to block 15. Similarly, the bimanual coordination factor (Bi-Co) improved in both groups *Long_C1* (Day 1): 44% (±14%, [5.7% to 52.3%]); *Long_C2*: 52% (±26%, [37% to 67%]). Finally, the evolution of forces exerted against the virtual walls (Bi-F) was not as uniform: in *Long_C1* (Day 1), the Bi-F improved with training *Long_C1* (Day 1): 16% (± 25%, [1% to 31%]), but there was a non-significant deterioration in *Long_C2* (*Long_C2*: -21% (±48%, [-49% to 7%]).

### Retention on Day 2

For the *Long_C1* group, the training continued on Day 2 with an additional 15 min of practice with the same circuit (C1) followed by a transfer test with circuit C3. Over the 2 days (*Equation 1)*, the Bi-SAT increased by 244% (±107, [181% to 307%]), the Bi-Co increased by 58% (±18%, [47% to 69%]) overall, and the Bi-F improved (i.e., decreased) by 22% (±19%, [11% to 33%]). After a large improvement on Day 1 in Bi-SAT: 148% (±73%, [105% to 191%]) and Bi-Co: 44% (±14%, [35.7% to 52.3%]), there was an overall large retention from Day 1 to Day 2, with a slight overnight loss from the last block of Day 1 to the first block of Day 2 (*Equation 1)* for the Bi-SAT (-10% (± 11%, [-16.5 % to -3.5%])) and Bi-Co (-3% (± 5%, [-%6 to 0%])). This loss which might have been due to the reaching movements at the end of Day 1 and the beginning of Day 2. Interestingly, the Bi-F improved by 122% (±21%,[110% to 134%]) from the last block of Day 1 to the first block of Day 2, indicating off-line improvement that was absent for the other two measures.

### Transfer to a New Circuit (Analysis 1)

To assess transfer after 15 and 30 min of training, both the *Long_C1* and *Long_C2* groups experienced a new circuit after training. For the *Long_C1*, the new circuit (C3) was introduced on Day 2, i.e., after a total of 30 min of training with C1. After large improvements with the trained circuit, performance with the new circuit deteriorated slightly (i.e., there was thus a large transfer) compared to the end of training with C1 in the Bi-SAT [-11%(±15%), *t*(10) = 2.45, *p* = 0.034, *d* = 0.6], there was no loss in the Bi-Co [-0.02% (± 4%), t(10) = -0.013, *p* = 0.99, *d* = 0.001] and improvement (decrease) in the Bi-F [12% (±16%), *t*(10) = 2.38, *p* = 0.038, *d* = 0.3]. These findings suggest that, after 30 min of training, ∼10% of the performance improvement was circuit-specific (i.e., there was an 11% loss in Bi-SAT at the transfer test), while the retained ∼90% was linked to the bimanual coordination control policy.

Furthermore, to test the transfer after 15 min of training with C2, we used the rotated version of C2 (namely, C1) as the new circuit. C1 required the performance of a new sequence of bimanual movements (circuit-specific skill), while the bimanual coordination policy remained unchanged. None of the subjects noticed that the circuit was “simply” rotated. Again, there was a decrease in performance when the circuit was changed after 15 min of training [Analysis 1: Bi-SAT: (-20% (±12%), *t*(10) = 5.45, *p* = 0.0003, *d* = 1.3); Bi-Co: (-6% (±3%), *t*(10) = 6.72, *p* < 0.0001, *d* = 1.19)] but no significant change in Bi-F: [11% (± 27%), t(10) = 1.35, p = 0.21, d = 0.4]. These findings suggest that ∼20% of the skill measured by Bi-SAT was circuit-specific after 15 min of training.

Thus, the circuit specificity was 20% after 15 min of training, but it was 10% after 30 min of training. However, we should consider that C1 and C3 were very similar (in Long_C1) (**Figure 1**), this is why the transfer was larger (90%).

### Training with Switching Circuits (Analysis 2)

**FIGURE 4:**
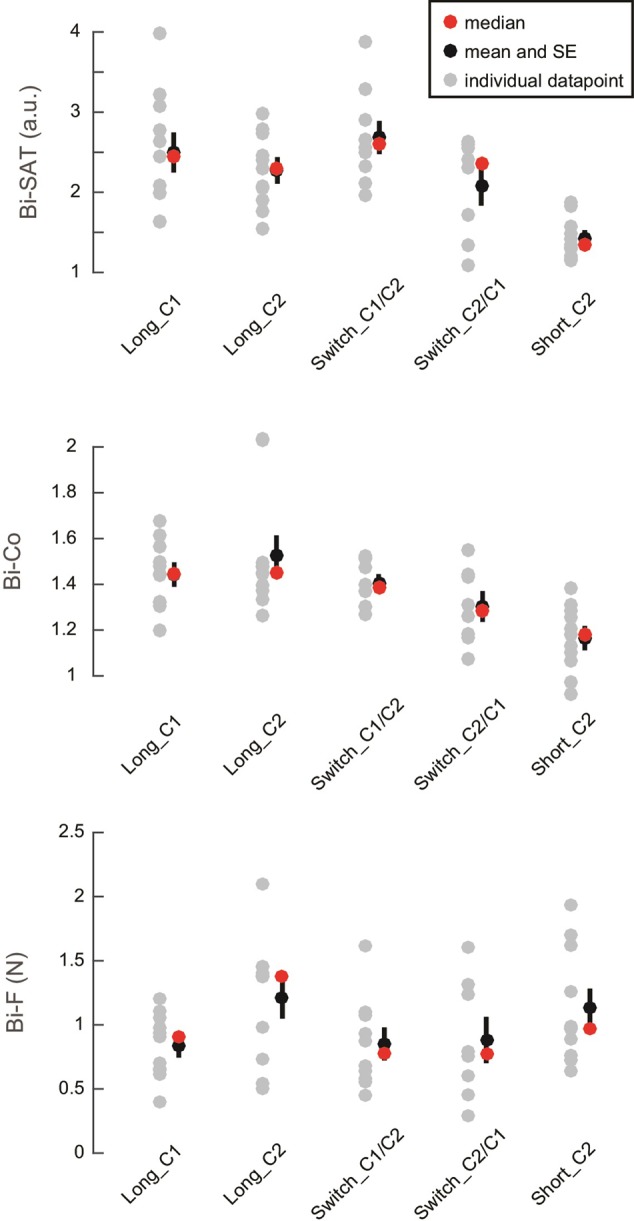
Change in the outcomes with different training regimens. Individual data points (in gray) are shown for the percentage of change between the 1st and last block of training in Bi-SAT **(top)**, Bi-Co **(middle)** and Bi-F **(bottom)**. The red dots represent medians and the black dots represent means with the black bars showing SEM. For Bi-SAT and Bi-Co, a change greater than 1 corresponds to improvement; for Bi-F, the percentage of change should be smaller than 1 as a *decrease* in the non-desired force reflects performance improvement.

To distinguish the learning of the bimanual coordination control policy (i.e., the general ability to apply bimanual coordination in this environment, irrespective of the circuit) from circuit-specific aspects (i.e., the specific sequence of bimanual movements needed to perform each circuit), two additional groups of subjects received 15 min of training while the C1 and C2 circuits were switched (Switch groups: *Switch_C1/C2* and *Switch_C2/C1*). By the end of training, these subjects achieved similar levels of performance (**Figure 4**) as subjects who learned with a single circuit (Long groups: *Long_C1* and *Long_C2*). To investigate the effect of the type of training (factor schedule: with the same circuit (Long) vs. with two switching circuits (Switch)) and the effect of the initial circuit of training (C1 vs. C2), a 2 × 2 ANOVA (Long vs. Switch) and (starting with C1 vs. starting with C2) was performed. There was no systematic difference between these two schedules for (Long vs. Switch), as the effects of schedule varied among the analyzed outcome. The Bi-Co was better in the Long groups than in the Switch groups [Analysis 2 – main effect of schedule: *F*(1,36) = 5.23; *p* = 0.028, η^2^= 0.12], while there was no difference between the schedules in the Bi-SAT [main effect of group: *F*(1,36) = 0.0003; *p* = 0.98, η^2^< 0.001] or in the Bi-F [main effect of group: *F*(1,36) = 1.6; *p* = 0.21, η^2^= 0.04]. There was an interaction between the factors “schedule” and “initial circuit” for the Bi-SAT [*F*(1,36) = 4.73; *p* = 0.036, η^2^= 0.11], but no interaction was found for the Bi-Co [*F*(1,36) = 0.0008; *p* = 0.88, η^2^< 0.001] or the Bi-F [*F*(1,36) = 2.6; *p* = 0.11, η^2^= 0.06]. For completeness, we report the effect of schedule, the starting circuit (C1 vs. C2) and the interaction between them in **Table 1**. Overall, these results demonstrated that switching between two different circuits did not strongly influence the ability to learn the bimanual coordination task, which suggests that most of the learning was related to the bimanual coordination control policy and not to the learning of the circuit-specific aspects (i.e., the sequence of (bimanual) movements needed to run the cursor across a specific order of segments in a given circuit).

**Table 1:** Analysis 2: Effect of training schedule (Long vs. Switch), starting circuit (C1 vs. C2) and their interaction on the three outcomes of learning.

	Long vs. Switch	C1 vs. C2 as first circuit	interaction
Bi-SAT	*F*(1,36) = 0.0003; *p* = 0.98	*F*(1,36) = 0.99; *p* = 0.32	*F*(1,36) = 4.73; *p* = 0.036
Bi-Co	*F*(1,36) = 5.23; *p* = 0.028	*F*(1,36) = 2.59; *p* = 0.12	*F*(1,36) = 0.0008; *p* = 0.88
Bi-F	*F*(1,36) = 1.6; *p* = 0.21	*F*(1,36) = 1.9; *p* = 0.17	*F*(1,36) = 2.6; *p* = 0.11

#### Performance in C1 vs. C2 (Analysis 3)

**FIGURE 5:**
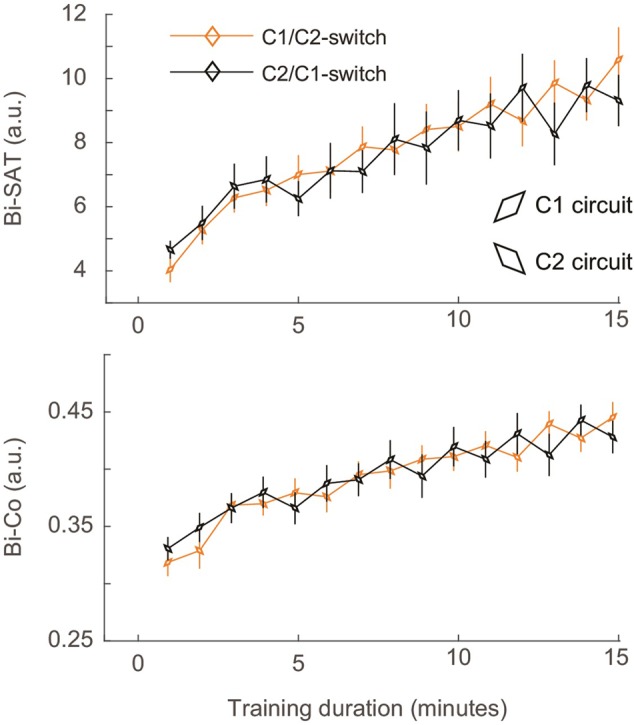
Evolution of the mean in Bi-SAT and Bi-Co for the Switch groups. Data from the *Switch_C1/C2* is represented by the orange curve and that of the *Switch_C2/C1* by the black curve. The angle of the diamond illustrates the circuit used on a particular block. Error bars represent SEM.

The result for the Bi-SAT and Bi-Co in the *Switch_C1/C2* and *Switch_C2/C1* groups revealed an oscillation in performance that began at block 5 (**Figure 5**). In both *Switch_C1/C2* and *Switch_C2/C1* groups, the performance on C2 was consistently lower than the performance on C1 [Analysis 3 - main effect of circuit for Bi-SAT: *F*(1,16) = 29.76, *p* < 0.0001, η^2^= 0.65; main effect of circuit for Bi-Co: *F*(1, 16) = 28.01, *p* < 0.0001, η^2^= 0.63; main effect of circuit for Bi-F: *F*(1, 16) = 6.31, *p* = 0.023, η^2^= 0.28]. This pattern resulted in oscillations in the values of Bi-Co and Bi-SAT because the circuits switched at each block. Despite this circuit switch from the beginning of training, there was no oscillation before block 5. Such oscillations were absent in the Bi-F (data not shown).

### Circuit-Specific Skill Arises Later during Learning (Analysis 4)

We postulated that the mentioned oscillations in the Bi-SAT and Bi-Co were due to a circuit-specific process. Because the oscillations arose only after block 5, we hypothesized that the first 5 min of training were mainly about the learning of the bimanual coordination control policy and were not/less about circuit-specific aspects. To test this, we ran an additional group (*Short_C2*) that trained for 4 min with the C2 circuit before experiencing C1 (to assess transfer) and compared the results to the transfer after training of the *Long_C2* group. For both of these groups, the training circuit was C2, and the transfer circuit was C1. The hypothesis was that when a new circuit was presented after only four blocks of training, there should be no decrease in performance if learning the bimanual coordination control policy dominated the early part of training. By contrast, the decrease in performance during transfer should be much larger after 15 min of training if circuit-specific skill provides the larger contribution.

**FIGURE 6:**
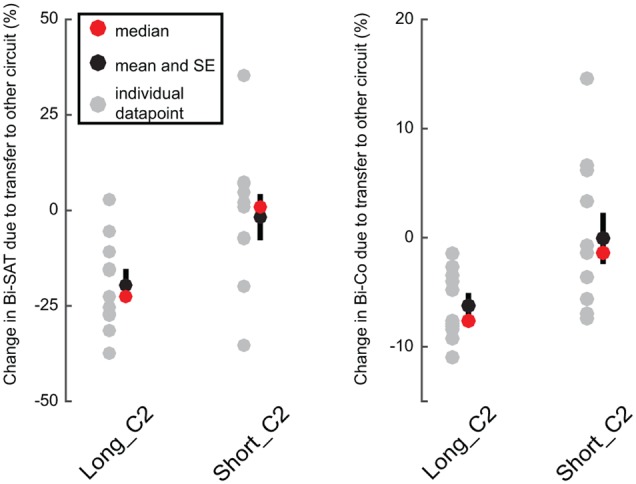
Performance drop after the circuit-switch in early vs. later phases of bimanual motor skill learning. Individual data points (in gray) are shown for the loss of performance (in %) between the last block of training and the transfer block (the blocks 15 and 16 for the *Long_C2* group and the blocks 4 and 5 for the *Short_C2* group). The red dots represent medians and the black dots represent means with the black bars showing standard error of the mean. Note that the scale is different for Bi-SAT and Bi-Co.

As expected, there was very little decrease due to the transfer test (i.e., P_EoL_) between the last block of training (block 4) and the transfer block (block 5) in *Short_C2* [Bi-SAT: -2% (±18%); Bi-Co: -0.01%(±7%)]. In comparison, there was a larger drop (i.e., P_EoL_) [Bi-SAT: -19% (± 9%); Bi-Co: -6% (± 2%)] after 15 min of training, i.e., between the last block of training (block 15) and the transfer block (block 16) in *Long_C2* (**Figure 6**): for Bi-SAT [*t*(20) = 2.77, *p* = 0.012, *d* = 1.4] and Bi-Co [*t*(20) = 2.69, *p* = 0.014, *d* = 1.4; Analysis 4]. This effect remained significant even after adjusting the significance threshold for multiple comparisons (*p* < 0.0166). No difference was detected in the Bi-F [*t*(20) = 0.39; *p* = 0.7, *d* = 0.3] in the *Short_C2* [Bi-F: -6% (±19%)] or in the *Long_C2* [Bi-F: -10%(±23%)].

### Correlation between the Three Outcomes Measures (Analysis 5)

We tested the correlation between the different outcomes. For each subject (Analysis 5, *N* = 40), we measured the performance in the 15th minute of training. We found a consistent correlation between the Bi-Co and Bi-SAT (*R* = 0.46, *p* = 0.0027), but not between Bi-F and either of the other two other variables (Bi-SAT vs. Bi-F: *R* = -0.22, *p* = 0.17, Bi-Co vs. Bi-F: *R* = -0.074, *p* = 0.65).

## Discussion

In this study, healthy individuals learned a new bimanual cooperative task. Subjects first acquired the bimanual coordination control policy and later learned circuit-specific (task-specific) aspects. With the current task, circuit-specific skill became more prominent with more advanced training and represented approximately 20% of the improvement in the Bi-SAT after 15 min of training.

We designed a new bimanual cooperative task in which a constant bimanual coordination control policy is necessary to perform the task. Three outcomes (Bi-SAT, Bi-Co and Bi-F) were defined to explore different aspects of bimanual performance. Increases in the Bi-SAT quantified the overall learning of the bimanual skill, which was expressed by a shift in the speed/accuracy trade-off (SAT) (Dayan and Cohen, 2011; Krakauer and Mazzoni, 2011; Reis et al., 2017) of the common cursor displacements. This increase was the result of either of the two following possibilities: (i) a concomitant improvement of both speed and accuracy, or (ii) an improvement of one parameter without concomitant deterioration of the other. By contrast, the Bi-Co quantifies how well the subjects coordinate their hands. The ideal Bi-Co occurs when the velocities of both hands are perfectly correlated and contribute equally to the common cursor displacement. The consistent correlation between the Bi-SAT and Bi-Co evolutions suggests that these measures may partly overlap in reflecting the same process. Indeed, the Bi-SAT is influenced by the quality of the bimanual coordination control policy and by the speed and accuracy of the cursor resulting from this coordination. We used three outcomes to quantify the subjects’ performance on the circuit. However, based on our design, the amount of circuit-specific skill is best characterized by the Bi-SAT outcome since it takes the error into account (which is highly dependent of the form of the circuit). Clearly, Bi-Co is also partially sensitive to the change in circuit but the drop is larger in Bi-SAT, that is why Bi-SAT was used to quantify how much of the improvements were related to circuit-specific skill during the training (i.e., after 15 and 30 min of training). In addition, although C2 and C1 had an identical shape (but rotated), all subjects found C2 more difficult and their performance was better on C1 to different extents in the two schedules (see **Table 1**). Given that Bi-SAT was highly related to the shape of the circuit, the difference in difficulty between C1 and C2 might be minimized when the same circuit is presented repeatedly compared to when the circuit switched, which might explain the significant interaction between circuit and schedule for that measure on the last block of training. Interestingly, the Bi-F evolved independently from the two other variables and might thus reflect a different process. With the current experimental protocol, while instructions and feedback signals focused on improving bimanual coordination through SAT, there was neither instruction nor penalty in the case of exerting forces against the virtual walls. Yet, the natural tendency of human to minimize the amount of energy use led to a decrease in the force exerted in the task-irrelevant directions (Diedrichsen et al., 2010).

In this new bimanual cooperative task, there was very large overnight retention of performance. After 30 min of training over 2 days, performing the task with a new version of the circuit resulted in a very slight loss in performance, indicating a large generalization of the bimanual performance, which is another hallmark of motor skill learning (Schmidt, 1975; Shea and Wulf, 2005; Platz et al., 2012).

### Learning Bimanual Coordination vs. Learning the Shape of the Circuit

By switching the two circuits during early learning, we identified two processes of bimanual motor skill learning: a bimanual coordination control policy that appeared from the very beginning of training and represented approximately 80% of the performance improvement, and a circuit-specific process that became observable approximately 5 min later.

While Sailer et al. (2005) observed a first phase of exploration in search of the appropriate bimanual control policy without improvement in bimanual coordination; we did not observe such a phase, as we physically imposed the appropriate bimanual coordination. In our experiments, the first process of learning corresponded to the acquisition of a bimanual coordination control policy for the following reasons: (1) the oscillation in performance appeared only after five blocks of training in both *Switch* groups, and (2) the performance loss due to the circuit switch was negligible after only four minutes of training (**Figure 6**) compared to the performance loss after 15 min of training.

This learning of the bimanual coordination control policy is comparable to the second stage proposed by Fitts and Posner (1967) during which the appropriate motor plans have to be elaborated. In our bimanual task, this elaboration requires the acquisition of a new bimanual coordination control policy in which both hands move in synergy. That is, because the hands need to move at the same speed (but along different axes), any change in speed from one hand has to be complemented by a similar change in speed in the other hand. Therefore, learning this bimanual coordination task consisted of establishing an integrated bimanual coordination control policy.

Here, the circuit-specific process of bimanual performance improvement evolves from 0 to 20% of the learning in approximately 10 min of training; its contribution to the overall improvement remains smaller than that of the bimanual coordination control policy. We did not test the “automaticity” of the learned behavior (another hallmark of a well-mastered skill) because of the restricted amount of training. We hypothesize that automaticity should gradually appears and should be transferrable to other tasks once it is sufficiently automatized, likely after more extensive bimanual training. This should be tested in future studies.

Importantly, while Fitts and Posner (1967) and Sailer et al. (2005) determined the stages of learning based on the rate of change in performance (i.e., the change in the skill acquisition rate), we used a generalization task to identify the different processes of learning. We believe that this strategy refines the understanding of the processes underlying skill improvement.

### Perspectives

Individuals with neurological conditions may present difficulties in coordinating their hands and are consequently restricted in their daily activities. Neurological patients with unilateral hand impairment (e.g., those who have suffered from a stroke, a traumatic brain injury, or multiple sclerosis) are partially or fully impaired in bimanual tasks (McCombe Waller et al., 2006; Wu et al., 2009; Johnson et al., 2011). A crucial step to develop efficient neurorehabilitation strategies is to translate the various stages of skill learning associated with the acquisition of new bimanual coordination control policies into novel neurorehabilitation strategies. For example, our study suggests that if a therapist wants to maximize the learning of a bimanual coordination process and to minimize the amount of task-specific (circuit-specific in our case) learning, she/he should propose a variety of bimanual tasks to enhance the exploration of different bimanual coordination control policies and to reduce the amount of task-specific learning. Our study suggests that 5 min of training on a task is enough for young subjects to limit task-specific (circuit-specific in our study) learning. This time will probably be somewhat longer for older people (Salimpour and Shadmehr, 2014) or in patients. Yet, it is important to propose a large variety of tasks to ensure general improvement in bimanual coordination control policy.

Task variability in such paradigms could lead to contextual interference (CI) (Magill and Hall, 1990). Studies have demonstrated that the performance level for subjects who train using one task (blocked training) is higher at the end of training than in those who train with varying tasks (random training) (Pauwels et al., 2014). Yet, random training leads to better retention than blocked training. We did not observe differences in learning between random and blocked training (with only two different circuits). It remains possible that retention on Day 2 might have been larger in the Switch groups compared to the non-switch groups.

### Limitations

In this study, the baseline bimanual capacities of healthy individuals were not evaluated with standard neuropsychological tests of bimanual performance. It is therefore possible that the groups differed in the average individual differences in bimanual capacities. Additionally, we did not train our subjects to asymptote. Even after 30 min of training, performance was still improving. Therefore, we do not know whether the circuit-specific process of the learning could have become larger with more training. Moreover, the current findings are valid in our task in which two hands ideally have equal contributions, however further investigations are necessary to generalize these results to tasks with an unequal contribution of each hand (Sisti et al., 2011). Finally, this study was performed only with young subjects, and further studies are needed to investigate its validity for older individuals and in larger groups to confirm and refine these findings. Such investigation is essential to assess the validity of the theory that the bimanual motor task should be changed frequently during rehabilitation.

## Conclusion

When healthy individuals were trained with a new complex bimanual task that requires learning a novel bimanual coordination control policy in a non-rhythmic, cooperative mode, different processes of early bimanual motor skill learning were identified. During the first blocks of bimanual motor skill learning, acquisition of the new bimanual coordination control policy largely dominated, and later, with further training, the sequence-specific aspect appeared.

Together, these results demonstrate that early bimanual skill learning is supported by at least two different processes. The new bimanual coordination control policy is established very early during learning. After a few minutes, circuit-specific improvements develop in parallel with further improvements in the bimanual coordination control policy.

## Note

All data files are available from the Open Science Framework database: (doi: 10.17605/OSF.IO/8QM9E; URL: https://osf.io/8qm9e/).

## Author Contributions

MYD and J-JOdX: study design, data acquisition, data analysis, statistical analysis, and wrote the paper. BB: study design, data analysis, statistical analysis, and revised the paper. YV: study design, supervised and interpreted data analysis, and wrote the paper.

## Conflict of Interest Statement

The authors declare that the research was conducted in the absence of any commercial or financial relationships that could be construed as a potential conflict of interest.

## Abbreviations

Bi-Co: bimanual coordination
Bi-F: bimanual force in the non-desired directions
Bi-SAT: bimanual speed-accuracy trade-off
EoL: End-of-Learning
